# Healthcare procurement in the race to net-zero: Practical steps for healthcare leadership

**DOI:** 10.1177/08404704241258152

**Published:** 2024-07-21

**Authors:** Declan C. T. Lavoie, Anika Maraj, Gigi Y. C. Wong, Fiona Parascandalo, Myles Sergeant

**Affiliations:** 1 3710 McMaster University, Hamilton, Ontario, Canada.; 2 St. Michael’s Hospital, Toronto, Ontario, Canada.; 3 8167 Vancouver General Hospital, Vancouver, British Columbia, Canada.

## Abstract

Although it is challenging to assess the greenhouse gas emission footprint associated with individual products and services, health leaders can play a pivotal role in emissions reduction by understanding and utilizing available tools and certifications that measure suppliers’ operational environmental performance. Integrating environmental standards into procurement and supplier selection has the potential to greatly impact emissions production across the healthcare landscape as it will pressure suppliers to improve their operations in order to be selected. The purpose of this article is to emphasize the importance of the supply chain in addressing healthcare-related greenhouse gas emissions. We provide an overview of the types of tools available that can be used to evaluate the carbon footprints of individual companies and rate their performances, as well as certifications that formally recognize companies’ sustainability practices and commitments.

## Background

The healthcare sector is a significant contributor to global Greenhouse Gas (GHG) emissions; with Canada’s healthcare system emitting 5.1% of Canada’s national carbon footprint (per capital), 7.9% of the footprint in the United States, and 5.9% of Great Britain’s footprint.^1^ Within the healthcare sector, supply chains represent the largest contributor of GHG emissions.^2^ According to a study by the National Health Service (NHS),^3^ the supply chain represents 62% of the healthcare carbon footprint. The three most significant streams within the supply chain are pharmaceuticals, medical devices, and business services.^4^

Globally, healthcare sector spending now accounts for $9 trillion USD (nearly 11% of gross domestic product).^5^ The best option to reduce healthcare’s carbon footprint is to prioritize buying from the suppliers that are most committed to reducing their GHGs.^6^ Committed leadership can be an important factor in delivering an impactful “green” procurement practice within an organization.^7^ Pressure from purchasers can lead to changes by their suppliers and has been shown to be a catalyst for supplier uptake of GHG reporting tools.^8,9^ To this end, as a leader in the field, the NHS announced in 2022 that a minimum net-zero and social value weighting of 10% will be implemented in the evaluation criteria for all NHS procurements.^10^

Net-zero procurement measures are important in helping Canada and other nations in meeting their global commitments to address climate change as laid out in initiatives such as the Paris Agreement.^11^ COP28 also held roundtables emphasizing the need for changes in procurement to achieve net-zero goals.^12^ Healthcare procurement offices and leadership decisions in these areas are now being seen as harbingers of innovation with market-shaping capacity.^13^ Creating a sustainable supply chain means understanding the lifecycle of products, environmental risks of materials, energy efficiency of suppliers, and chemical safety, among other qualifiers.^14^ Accordingly, tools have been created to evaluate the carbon footprints and climate impact of individual companies and rate their performance.^15-17^ Use of these tools reduces the complexity of understanding emissions throughout the supply chain and bridges the knowledge gap that can be faced when implementing sustainable practices.

Our intention with this article is to have leaders and decision-makers understand the importance of net-zero procurement tools and certifications, and to inspire these leaders to use their considerable purchasing power to influence supplier practices. The tools covered fall into two categories: evaluation tools, which generate numeric or letter scores that can be compared; and certifications, which companies may obtain if they demonstrate adherence to specific science-based GHG emission reduction targets throughout the supply chain.

In our discussion of low-carbon procurement tools and certifications, we will be focusing on their usefulness within the supply chains of medications and devices. These are two of the largest contributors of GHGs within healthcare’s supply chain and their lifecycles integrate complex, often global systems. This complexity and the evolving nature of regulatory policies and global networks of producers makes management decisions an important factor in supply chains for medications and devices.^18^ Furthermore, shortages and supply disruptions in these areas are felt by patients and staff directly, as COVID-19 made apparent.^18,19^ Utilization of the available tools and certification in making these decisions could potentially add efficiency to the work of leadership and increase transparency in supply chains that are often opaque.^18,20^

## Evaluation tools

There are a number of organizations that assess GHG emission and their overall climate impact. We present two long-standing, international, and widely implemented corporate environmental sustainability systems: Science-Based Targets initiative (SBTi)^21^ and CDP (formerly Carbon Disclosure Project).^17^ These tools provide insight into the relative efforts and success of medication and medical device manufacturers in reducing their carbon emissions.

As an example of how these tools are used, Table 1 presents the scores of six anonymized companies. This table will help readers understand how to compare the tools discussed in this article. The companies included have achieved varying scores in each of these rating systems, with companies’ “A,” “B,” and “X” outperforming competitors in their respective industries.

**Table 1.** Six companies in healthcare-adjacent industries stratified according to environmental sustainability performance according to three rating systems.

			SBTi	CDP
Sector	Company	Near-term target	Target year	Near-term target Target year
Pharmaceuticals	A	1.5°C	2026	A-
	B	1.5°C	2030	A-
	C	No target set		C
Medical devices	X	1.5°C	2030	A-
	Y	1.5°C	2030	B
	Z	No target set		B-
Interpretation		The near-term target represents a company’s commitment to reduce GHG emissions in line with limiting global warming to 1.5° or 2° over 5-10 years. The target year reflects the timeline for achieving the near-term target		Participating companies are scored from D- (worst) to A (best)
Advantages		SBTi data set is open-source		CDP scores 23,202 companies worldwide as of 2023
		The methodology used to validate targets is fully transparent		
Disadvantages		Target setting and reporting is voluntary; only 2,569 companies have set targets with SBTi		Companies that do not self-report are assigned a score of F, and data collection can be challenging
		Some criticize the methodology of targets		Access to the full dataset requires a licence
Web link		https://sciencebasedtargets.org/companies-taking-action#dashboard		https://www.cdp.net/en/responses?queries%5Bname%5D=

### SBTi targets

The SBTi is a non-profit collaboration between CDP, the United Nations Global Compact, World Resources Institute, and World Wide Fund for Nature.^22^ They provide companies with tools to set realistic emissions reduction targets that align with current climate science.^1^ They also help companies develop concrete strategies to achieve those targets and monitor their progress on an annual basis. The SBTi maintains a freely available database of thousands of companies that have set or are committed to setting emissions reduction targets. Companies may set near-term, long-term, and net-zero targets. These targets are subsequently validated by SBTi, and progress reports are mandatory to ensure that companies remain on track to achieve them. Emerging evidence suggests companies that set science-based targets are, on average, setting more ambitious emissions reduction targets than companies that set internal targets, and many are on track to meet their emissions targets.^22^

There is some debate around the specific methodological choices that define SBTi’s targets, and some critics are calling for more engagement and scrutiny around their policies. The targets have been seen as narrow and only allowing for a specific idea of a decarbonized future.^23^ Additionally, as listed in Table 1, an important disadvantage is that companies set their own targets and could be incentivized to set targets they know they will meet, while avoiding more ambitious changes.^24^

Proponents of this tool find that it creates competition between users, driving change across industries and positively impacting corporate culture.^8^ A 2022 study found that firms setting targets with SBTi reduce their absolute GHG emissions by about 5% and their carbon intensity by 8%–10%. This result is not found for firms that did not set targets. Notably, the firms that reduced their emissions the most did so without deteriorating financial performance.^25^

### CDP

CDP is an international non-profit organization that has published a global environmental disclosure system since 2000.^2^ CDP compiles disclosures from companies, cities, states, and regions across more than 90 countries and uses this data to generate an annual list of CDP scores.^26^ Scores serve as a reflection of participating companies’ environmental performance and are meant to motivate corporate sustainability.^27^ Indeed, companies that participate in carbon disclosure do show improvements in carbon performance.^28^

Companies that participate in the CDP disclosure questionnaire are scored on four hierarchical levels: disclosure, awareness of environmental issues, management methods and progress towards environmental leadership.^27^ A minimum score on one level is required to be assessed at the next level. Scores are generated across three themes (climate change, which is displayed in Table 1, water security, and forests). The scoring itself is conducted by a CDP-trained scoring partner and subsequently quality checked by the CDP internal scoring team.^29^

As stated in Table 1, the number of participants in CDP is a key advantage.^30^ While companies do not have to disclose their emission information, the market can evaluate the reliability of the carbon emission information by comparing reported data across an industry, using companies that volunteer their data as benchmarks. This also means that pressure from purchasers to have suppliers report their emissions can add transparency, accuracy, and reliability to emissions data across an industry.^9,30^

Participants in CDP have reported that collecting accurate data on their emissions can be challenging, and this may affect reporting. This was especially true of companies with global operations.^9^

## Corporate certificates

Certifications brand suppliers as adhering to specific environment practices throughout their operations. The certifications detailed below are becoming standard within industry and are heavily discussed in the literature. We also highlight a Canadian specific certification which may be useful for Canadian organizations that prefer local supply chains.

### B Corporation

Amongst the many sustainability certifications available, one example with global participation is the B Corporation certification.^3^ B Lab, which administers the certification, is a global non-profit that aims to “make business a force for good.”^31^ Unlike the other certifications covered in this section, B Certification does not primarily focus scoring on emissions. The certification is awarded to companies that meet a high standard of performance in the domains of environment, governance, community, workers, and customers.^32^

One concern about the B corporation is that the complexity and cost of the certification process may deter smaller companies from participating. To date, B Corporations include 7,062 companies in 161 industries.^31^ Unfortunately, very few healthcare companies have obtained B Corporation certification. There are, however, many products purchased by healthcare organizations whose suppliers can be found in the list of B Corporations (e.g., food and clothing).

It can be difficult to identify socially and environmentally responsible companies, and B Corporation certification eases this process for procurement offices.^33^ Purchasing from suppliers with this certification signifies to stakeholders (e.g., staff, patients, and community) that the purchaser values social and environmental progress along with sound economic decisions.^34^ Consequently, as suppliers are aware their products are being purchased for more than just their material value, purchasers may be expected to pay a price premium.^35^

### UN race to zero campaign

The United Nations Framework Convention on Climate Change has spearheaded the Race to Zero (R2Z) campaign.^4^ R2Z aims to reduce global carbon emissions to zero by the year 2050 by establishing clear standards for net-zero emissions pledges by non-state entities (e.g., corporations, cities, and financial, educational, and healthcare institutions).^36^ These entities may become R2Z members through officially recognized partners (of note, the SBTi is a R2Z partner).^37^

R2Z uses two sets of criteria—“Starting Line,” and “Leadership Practices.” The Starting Line criteria outline the minimum requirements that members much achieve to join the campaign, including developing a plan to reduce GHG emissions to net-zero by the year 2050. Targets must address the emissions directly associated with company operations as well as the supply chain. Leadership Practices are example pathways that members can follow to demonstrate exceptional commitment to climate action. R2Z currently has over 12,000 members, of which over 7,000 are companies.^36^

As governments move to integrate zero emission targets into their policies, there has been a proliferation of methods suppliers can use to indicate they are net-zero or working towards it. R2Z's global recognition and uptake, along with a high level of rigour, helps purchasers parse through the market.^38^

The central disadvantage of this certification is that participants in R2Z may have started the net-zero shift without necessarily being on track to be net-zero.^37^ This necessitates a degree of investigation on the purchasers’ side to see where the R2Z certified supplier is in the net-zero transition process.

### Canadian net-zero challenge

The Canadian government launched the Net-Zero Challenge in 2022.^5^ This initiative aims to help businesses transition their facilities and operations to net-zero emissions by 2050.^39^ Participating companies must set a credible net-zero GHG target for 2050 or earlier, develop credible plans to achieve interim targets, and report progress annually.^40^ To date, 107 companies with operations in Canada have joined the challenge.^39^

Regulations such as the Building Ontario Businesses Initiative (BOBI) mandate establishing local supply chains. Canadian focused net-zero certifications could be helpful tools when making environmentally conscious shifts from a global market. The certification is not widely used currently, but this may change as regulations continue to prioritize local supply chains.

## Discussion and recommendations

It is the authors’ recommendation that leaders and decision-makers within healthcare organizations strive to integrate sustainable practices into procurement policies. Since procurement makes up a large percentage of healthcare GHG emissions, shifts in how suppliers are evaluated and selected has the potential to lead to shifts in supplier operations and potentially shape the market.^8,13^ The tools and certifications presented here may help leadership gain a comprehensive understanding of a supplier's GHG reduction plans and can reduce the risk of working with suppliers that are not preparing their operations for the consequences of climate change.^30^ As healthcare related emissions are being increasingly recognized and healthcare organizations are beginning to take action, procurement is an important area of focus.^8,9^ To avoid the worst outcomes of climate change, global action is needed by 2050 and healthcare needs to take serious steps in addressing emission contributions.^41^

The cost of shifting practices and engaging new suppliers is a barrier in implementing sustainable procurement. In publicly funded organizations, such as healthcare, this can be one of the largest barriers to making the shift.^42^ Lack of leadership involvement and support is also a leading barrier,^42^ making it even more important that leaders are aware of the options and the benefits they will bring to their organizations’ GHG footprint.

There is a need for more research into the impacts, barriers, and facilitators of integrating the tools and certifications we included. Case studies within Canadian healthcare settings could be useful for the sector as a whole. Specifically, the costs associated with shifting policies and establishing new supplier relations with organizations that use and perform well with these tools and certifications should be investigated. With the fiscal constraints inherent to the healthcare system, understanding all the costs and savings involved is essential. Lastly, additional research to determine the most effective tool for healthcare facilities and studies on effective implementation practices for these tools in different healthcare settings (i.e., hospital vs. family clinic) are needed.

## Conclusions

When we purchase products, we not only acquire the products themselves but also the GHG emissions associated with their production and transportation. The healthcare procurement process involves a number of decision-makers, including clinicians, surgeons, facilities managers, food managers, procurement leads, chief financial officers, and group purchasing organizations. Leaders in all of these segments have the responsibility to prioritize buying from the companies doing the most to reduce their GHG emissions. By purchasing from companies that have demonstrated a commitment to net-zero GHG emission targets, they signal to industries that addressing climate change is a key consideration in procurement decisions. In turn, companies will be motivated to reduce their carbon footprints to remain competitive in their respective markets. Furthermore, a shift to net-zero procurement models and taking part in initiatives such as the UN Race to Zero campaign has the potential to fundamentally shift supplier practices so that they take on their own commitments to science-based net-zero targets.^43^

Corporate net-zero rating and certification systems are available to assist health leaders in selecting companies with clear commitments to environmental stewardship. Net-zero procurement is a key strategy for achieving net-zero carbon emissions in the healthcare sector.

## Notes

1 For more information on SBTi go to: https://sciencebasedtargets.org/companies-taking-action. View companies using SBTi: https://sciencebasedtargets.org/sectors

2 For more information on CDP, go to: https://www.cdp.net/en. To view CDP scores by enrolled companies, go to: https://www.cdp.net/en/scores

3 For more information on B Corp Global web site, go to: https://www.bcorporation.net/en-us/. To search for B Corp certified companies, go to: https://www.bcorporation.net/en-us/find-a-b-corp/

4 For more information about web site: https://www.un.org/en/climate-action/race-net-zero-economy. To search for List of participants: https://tinyurl.com/bdfcn4cy

5 For more information about web site: https://www.canada.ca/en/services/environment/weather/climatechange/climate-plan/net-zero-emissions-2050/challenge.html. To search for list of participants: https://www.canada.ca/en/services/environment/weather/climatechange/climate-plan/net-zero-emissions-2050/challenge.html#participants
